# Follicle-Stimulating Hormone and Testosterone Play a Role in the Regulation of Sertoli Cell Functions Following Germ Cell Depletion In Vitro

**DOI:** 10.3390/ijms26062702

**Published:** 2025-03-17

**Authors:** Alaa Sawaied, Bat-El Levy, Eden Arazi, Eitan Lunenfeld, Qinghua Shi, Mahmoud Huleihel

**Affiliations:** 1 The Shraga Segal Department of Microbiology, Immunology and Genetics, Faculty of Health Sciences, Ben-Gurion University of the Negev, Beer Sheva 84105, Israel; alaasa@post.bgu.ac.il (A.S.); batelll@post.bgu.ac.il (B.-E.L.); edenaraz@post.bgu.ac.il (E.A.); 2 The Center of Advanced Research and Education in Reproduction (CARER), Faculty of Health Sciences, Ben-Gurion University of the Negev, Beer Sheva 84105, Israel; 3 Adelson School of Medicine, Ariel University, Ariel 4070000, Israel; eitanlun@ariel.ac.il; 4 Centre for Reproduction and Genetics, First Affiliated Hospital of USTC, Biomedical Sciences and Health Laboratory of Anhui Province, School of Basic Medical Sciences, Division of Life Sciences and Medicine, Institute of Health and Medicine, Hefei Comprehensive National Science Center University of Science and Technology of China, Hefei 230000, China; qshi@ustc.edu.cn

**Keywords:** germ cell depletion, spermatogenesis, Sertoli cell activity in vitro, FSH, testosterone, Sertoli cell–germ cell interaction

## Abstract

Spermatogenesis is a process of self-renewal of spermatogonial stem cells and their proliferation and differentiation to generate mature sperm. This process involves interactions between testicular somatic (mainly Sertoli cells) and spermatogonial cells at their different stages of development. The functionality of Sertoli cells is regulated by hormones and testicular autocrine/paracrine factors. In this study, we investigated the effects of follicle-stimulating hormone (FSH) and testosterone addition on Sertoli cell cultures that undergo hypotonic shock, with a primary focus on Sertoli cell activity. Cells were enzymatically isolated from testicular seminiferous tubules of 7-day-old mice. These cells were cultured in vitro for 3 days. Thereafter, some cultures were treated with hypotonic shock to remove germ cells. After overnight, fresh media without (control; CT) or with FSH, testosterone (Tes), or FSH+T were added to the hypotonic shock-treated or untreated (CT) cultures for 24 h. The morphology of the cultures and the presence of Sertoli cells and germ cells were examined. The expression of growth factors (CSF-1, LIF, SCF, GDNF) or other specific Sertoli cell factors [transferrin, inhibin b, androgen receptor (AR), androgen binding protein (ABP), FSH receptor (FSHR)] was examined by qPCR. Our immunofluorescence staining showed depletion/major reduction in VASA-positive germ cells in Sertoli cell cultures following hypotonic shock (HYP) treatment compared to untreated cultures (WO). Furthermore, the expression of the examined growth factors and other factors was significantly increased in HYP cultures compared to WO (in the CT). However, the addition of hormones significantly decreased the expression levels of the growth factors in HYP cultures compared to WO cultures under the same treatment. In addition, the expression of all other examined Sertoli cell factors significantly changed following HYP treatment compared to WO and following treatment with FSH and or T. However, the expression levels of some factors remained normal following the treatment of Sertoli cell cultures with one or both hormones (transferrin, Fsh-r, Abp, Ar). Thus, our results demonstrate the crucial role of germ cells in the functionality of Sertoli cells and the possible role of FSH and T in maintaining, at least partially, the normal activity of Sertoli cells following germ cell depletion in vitro by hypotonic shock treatment.

## 1. Introduction

Male infertility affects approximately 20% of couples worldwide and has been linked to a variety of different causes [1]. Spermatogenesis is a complicated process that involves the proliferation and differentiation of spermatogonial stem cells (SSCs) to generate mature sperm [2,3,4,5]. This process occurs in the seminiferous tubules that contain the somatic cells (Sertoli cells, and peritubular cells) in addition to spermatogonial cells at different stages of development (meiotic and post-meiotic) [2,3,4,5]. FSH is produced by the pituitary gland and is involved in regulating the production of sperm through its effect on Sertoli cells [2,3,4,5]. Sertoli cells produce various factors following FSH stimulation such as androgen binding protein (ABP), androgen receptor (AR), transferrin, and inhibin b [4,5,6,7,8,9]. In addition, Sertoli cells produce different growth factors such as stem cell factor (SCF), colony-stimulating factor-1 (CSF-1) and glial-derived nerve growth factor (GDNF) [7,9,10,11,12]. Some of the factors produced by Sertoli cells were also shown to be produced by testicular germ cells including CSF-1, GDNF, granulocyte-macrophage colony-stimulating factor (GM-CSF), interleukin-1 family, and others [9,10,11,12,13,14,15,16]. These factors affect the proliferation and differentiation of spermatogonial stem cells to generate sperm. Testosterone, the primary male sex hormone, is produced by Leydig cells in response to luteinizing hormone (LH), which is required for the development of sperm [12,17]. Testosterone is necessary for a critical process in spermatogenesis, supporting the completion of meiosis, differentiation, and proliferation of spermatogonia stem cells, elongated sperm adhesion to Sertoli cells, Sertoli cell proliferation, and sperm release [3,17]. For normal spermatogenesis, cell–cell interactions between Sertoli cells and SSCs, at different stages of development, are crucial [4,5,9,10,18,19,20,21]. Depletion of spermatogonial cells was shown to disrupt the production of normal levels of factors produced by Sertoli cells [18,19,20]. Under chemotherapy treatments, in which testicular germ cells are mainly damaged and depleted [22,23,24], the activity of Sertoli cells is changed [19,22]. This is shown in the expression levels of various factors secreted by the Sertoli cells that are involved in spermatogenesis regulation [19,22,25].

Under normal conditions, FSH, testosterone, and Sertoli cell interactions are physiological. The dynamic balance between those components is integral to normal sperm production. An imbalance or alteration in the interaction between these hormones and testicular cells can create a situation where normal sperm production is disrupted, which may lead to male infertility [3,15,17,26,27].

FSH/LH-deficient mice showed germ cell developmental impairments, which caused infertility in those mice [15]. Sertoli cell counts were significantly lower in FSH/FSHR-deficient mice compared to normal mice [28]. FSH is important to preserve germ cells. FSH-deficient mice developed germ cells only in the spermatocyte stage [29]. FSH is essential for Sertoli cell division, induces Sertoli cells to secrete adhesion molecules, and is essential in Sertoli cell development [3,5,26,28,30].

LH affects the ability of Leydig cells to secrete testosterone. Blocking LH receptors caused a development impairment of the spermatogenesis process including sperm development [28,29,30].

Sertoli cells are in physical contact with testicular germ cells at their different stages of development; they also secrete molecules with signals that involve the control of the division and differentiation of germ cells such as transferrin, inhibin, and other growth factors [3,6,31]. Probably due to the proximity between Sertoli cells and germ cells, germ cells also control the activity of Sertoli cells [5,18,19,22].

Chemotherapy and radiotherapy treatments affect dividing cells, including those in the testes. It was shown that gonadotoxic treatments lead to germ cell depletion and impairment of somatic cell activities and impaired male fertility [19,20,23]. This study aimed to find out the effect of testicular germ cell depletion on the expression levels of growth factors and certain specific factors of Sertoli cells origin (that are involved in the development of normal spermatogenesis) under in vitro culture conditions. Furthermore, the study aims to evaluate the role of FSH and testosterone in the restoration of those factors.

## 2. Results

### 2.1. Characterization of the Cells Present in Cultures Before and After Hypotonic Shock In Vitro

**Figure 1:**
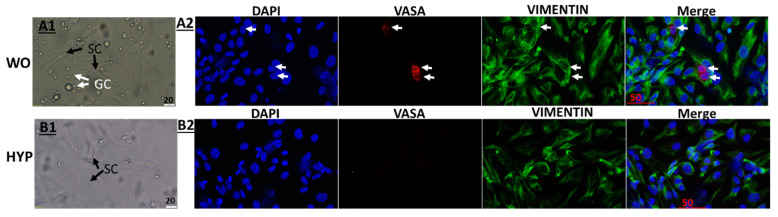
Characterization of the cells present in cultures before and after hypotonic shock in vitro. Cells were isolated and cultured as described in the materials and methods section. In the cultures without hypotonic shock (WO), we could identify the presence of adherent cells (Sertoli cells; SC) and germ cells (GC) (**A1**). White arrows indicate the location of VASA-positive cells in the culture. However, in the cultures treated with hypotonic shock (HYP), we could identify mainly Sertoli cells and individual germ cells (**B1**). Immunofluorescence staining of cultured WO, using anti-VIMENTIN antibodies (specific for Sertoli cells; green color) and anti-VASA antibodies (red color), showed the presence of both types of cells (**A2**), while HYP cultures showed only the presence of Sertoli cells (**B2**). Panels (**A1**,**B1**,**A2**,**B2**) do not correspond to the same location in the cell culture.

Isolated cells from seminiferous tubules of immature mice were cultured for 3 days followed by hypotonic shock (HYP) treatment or without hypotonic shock treatment (WO) as described in the materials and methods section. In cultured WO, we saw the presence of adherent cells (elongated) and non-adherent cells (round) after 24 h of adding fresh medium into the cultures (Figure 1A1). On the other hand, in cultures with HYP treatment, we saw mainly adherent (elongated) cells without or with very few round (non-adherent) cells (Figure 1B1).

To identify the types of adhered cells (supposed to be mainly Sertoli cells), we used immunofluorescence staining to stain the cultures WO and after HYP treatments with a specific marker for Sertoli cells (Vimentin) and DAPI dye (blue) to mark the cell nuclei. Our results show that most of the adherent cells in both cultures (WO and HYP) were positively stained for VIMENTIN (green color) (Figure 1A2,B2, respectively). To identify the non-adherent cells, we double-labeled the cultured WO and HYP-treated cells with a specific marker for premeiotic cells (VASA, red color) and Sertoli cells (VIMENTIN)) (green color). Our results show the presence of cells that were positively stained for VASA (red color) (10 ± 1%); and they were located between Sertoli (green color) in WO cultures, as expected (Figure 1A2). However, in cultures that were treated with HYP, we identified a very low number of cells with positive staining for VASA cells (red color) (3 ± 1%), and the majority of the cells were stained for Sertoli cells (green color) (Figure 1B2).

### 2.2. Expression of Different Factors in Cultured Sertoli Cells

**Figure 2:**
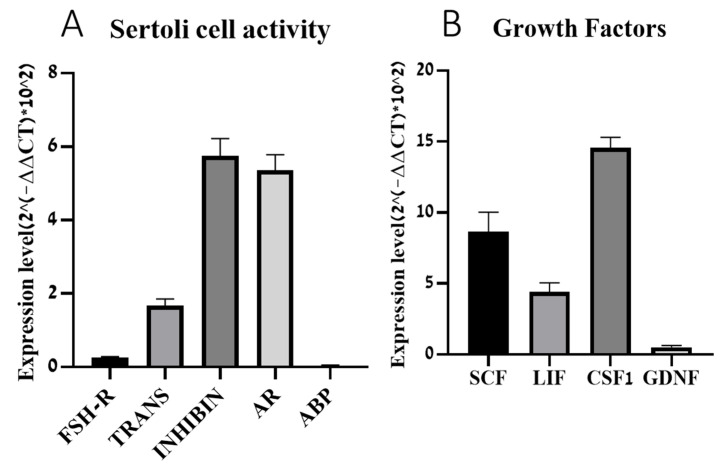
Expression of different factors in Sertoli cell cultures. Cells were isolated and cultured as described in the materials and methods section without hypotonic shock and hormone treatment. RNA was extracted from the cultures and the expression levels of factors that specifically indicate Sertoli cell activity (Fsh-r, Trans, Inhib b, Ar, Abp) (**A**), and growth factors produced by Sertoli cells (Scf, Lif, Csf-1, Gdnf) (**B**) were evaluated by qPCR analysis using specific primers for each factor. The expression levels were correlated to GAPDH of the same examined sample. N (the number of repetitions) = 3–4; n (the number of wells in each culture) = 2.

Isolated cells from seminiferous tubules of immature mice were cultured for 3 days without (WO) hypotonic shock treatment as described in the Materials and Methods section. Thereafter, fresh media were added overnight. RNA was extracted from those cultures and examined for the expression levels of different factors by qPCR analysis. The expression levels of the factors that are produced by Sertoli cells and indicate their activity such as FSH receptor (Fsh-r), transferrin, inhibin b, androgen receptor (Ar), and androgen binding protein (Abp) are presented in Figure 2. Our results show distinct expression levels of the examined factors. Transferrin, inhibin, and Ar showed high expression, while the expression levels of Fsh-r and Abp were relatively low (Figure 2A). In addition, these cultures expressed growth factors such as colony-stimulating factor-1 (Csf-1), leukemia inhibitory factor (Lif), stem cell factor (Scf), and glial cell-derived neurotrophic factor (Gdnf) (Figure 2B).

### 2.3. Effect of FSH and Testosterone In Vitro on the Expression of Factors Produced by Sertoli Cells

**Figure 3:**
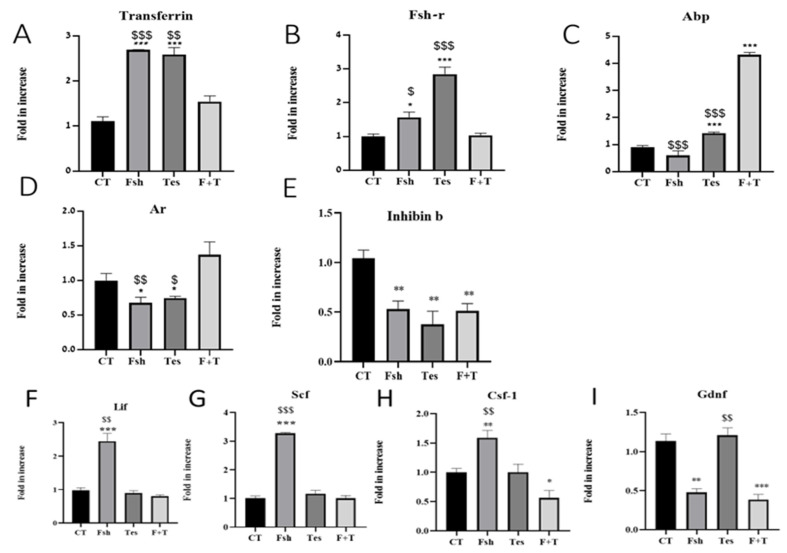
Effect of FSH and testosterone in vitro on the expression of factors produced by Sertoli cells. Cells were isolated and cultured as described in the Materials and Methods section without hypotonic shock (WO) in the absence or presence of FSH or testosterone (TES) or both (F + T). RNA was extracted from the cultures and the expression levels of factors that specifically indicate Sertoli cell activity (Trans, Fsh-r, Abp, Ar, inhibin b, Lif, Scf, Csf-1, and Gdnf) were evaluated by qPCR analysis using specific primers for each factor (**A**–**I**). N (the number of repetitions) = 3–4; n (the number of wells in each culture) = 2. *—compared to CT. * *p* < 0.05; ** *p* < 0.01; *** *p* < 0.001. $—compared to F + T. ^$^ *p* < 0.05; ^$$^ *p* < 0.01; ^$$$^ *p* < 0.001.

Isolated cells from seminiferous tubules of immature mice were cultured for 3 days without (WO) hypotonic shock treatment as described in the Materials and methods section. Thereafter, fresh media in the absence or presence of FSH (7.5 IU/mL), testosterone (TES; 10^−7^ M), or both hormones FSH and TES (F + T) were added overnight. The expression levels of factors that are specifically produced by Sertoli cells Fsh-r, Ar, transferrin, Abp, and inhibin b (Figure 3A–E), as well as factors that are secreted by Sertoli cells and other cells in the testicular tissue and are involved in controlling the division and differentiation of the germ cells such as Csf-1, Lif, Scf and Gdnf (Figure 3F–I), were examined in these cultures by qPCR analysis.

Our results show that the addition of FSH or TES significantly increased the expression level of transferrin (*p* < 0.001) and Fsh-r (*p* < 0.05 and *p* < 0.001, respectively) compared to the control (CT) (Figure 3A,B, respectively). However, the addition of both F + T did not affect their expression levels compared to CT (Figure 3A,B, respectively).

On the other hand, the addition of FSH did not affect the expression levels of Abp in these cultures compared to CT (Figure 3C). However, the addition of TES or F + T to these cultures significantly (*p* < 0.001) increased the expression levels of Abp compared to CT (Figure 3C). Furthermore, the addition of FSH or TES, but not both (F + T), to these cultures significantly (*p* < 0.05) decreased their capacity to express Ar compared to CT (Figure 3D). In addition, the addition of FSH, TES or both (F + T) to these cultures significantly (*p* < 0.01) decreased their capacity to express inhibin b compared to CT (Figure 3E).

The addition of testosterone (TES) to Sertoli cell cultures did not affect their capacity to express Lif, Scf, Csf-1 or Gdnf compared to CT (Figure 3F–H). However, the addition of FSH to Sertoli cell cultures significantly increased their capacity to express Lif (*p* < 0.001), Scf (*p* < 0.001), and Csf-1 (*p* < 0.01) but significantly decreased the expression levels of Gdnf (*p* < 0.01) compared to CT (Figure 3F–H and Figure 3I, respectively). On the other hand, the addition of both hormones (TES + FSH) to Sertoli cell cultures did not affect the expression levels of Lif and Scf compared to CT, but it significantly decreased their expression compared to FSH alone (Lif and Scf *p* < 0.001 and Csf-1 and Gdnf *p* < 0.01) (Figure 3F,G). However, the addition of both hormones to Sertoli cell cultures significantly decreased the expression levels of Csf-1 (*p* < 0.05) and Gdnf (*p* < 0.001) compared to CT and also Csf-1 compared to FSH alone and Gdnf compared to TES alone (Figure 3H,I).

### 2.4. Effect of Germ Cell Removal (Hypotonic Shock) and Hormones on the Expression of Factors Produced by Sertoli Cells In Vitro

Isolated cells from seminiferous tubules of immature mice were cultured for 3 days without (WO) or after hypotonic shock (HYP) treatment as described in the Materials and Methods section. Thereafter, fresh media in the absence or presence of FSH (7.5 IU/mL), testosterone (TES; 10^−7^ M), or both hormones FSH and TES (F + T) were added overnight. We compared the expression levels of factors specific to Sertoli cells such as transferrin, inhibin b, Ar, and Abp, in addition to growth factors such as Csf-1, Lif, Scf, and Gdnf in cells from HYP to WO cultures.

**Figure 4:**
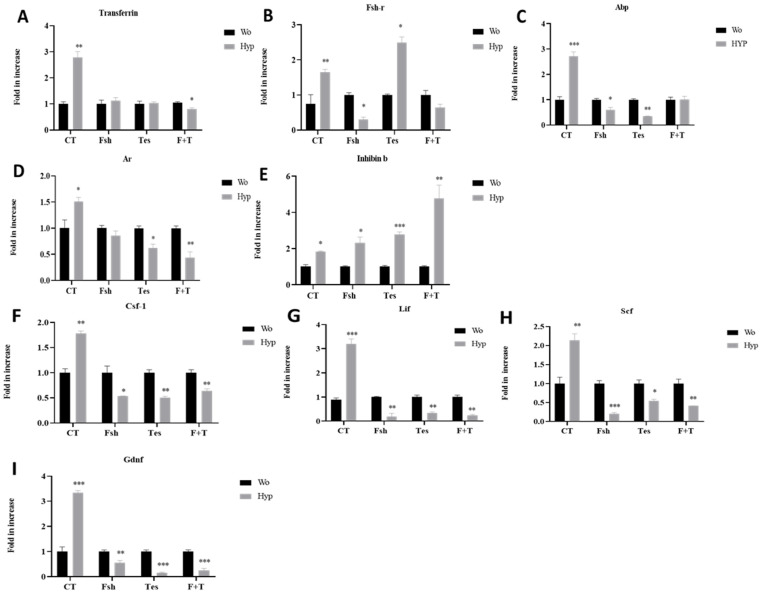
Effect of germ cell removal (hypotonic shock) and hormones on the expression of factors produced by Sertoli cells in vitro. Cells were isolated and cultured as described in the materials and methods section without (WO) and following hypotonic shock (HYP) in the absence or presence of FSH or testosterone (TES) or both (F + T). RNA was extracted from the cultures and the expression levels of factors that specifically indicate Sertoli cell activity (Trans, Fsh-r, Abp, Ar, Inhibin b, Csf-1, Lif, Scf, and Gdnf) were evaluated by qPCR analysis using specific primers for each factor (**A**–**I**). N (the number of repetitions) = 3–4; n (the number of wells in each culture) = 2. * comparison of Hyp (hypotonic shock) to the same treatment in Wo (without hypotonic shock) cultures. * *p* < 0.05; ** *p* < 0.01; *** *p* < 0.001.

Our results show that hypotonic shock (HYP) treatment significantly increased the expression level of transferrin (*p* < 0.01), Fsh-r (*p* < 0.01), Abp (*p* < 0.001), Ar (*p* < 0.05) and inhibin b (*p* < 0.05) and the growth factors Csf-1 (*p* < 0.01), Lif (*p* < 0.001), Scf (*p* < 0.01), and Gdnf (*p* < 0.001) after 24 h of incubation in cultures, compared to the WO treatment in the absence of hormones (CT) (Figure 4A–I, respectively, and Table 1). However, the addition of FSH to HYP-treated cultures did not affect the expression levels of transferrin and Ar and significantly increased the expression levels of inhibin b (*p* < 0.05), but significantly decreased the expression levels of Abp (*p* < 0.05), Fsh-r (*p* < 0.05), and the growth factors Csf-1 (*p* < 0.05), Lif (*p* < 0.01), Scf (*p* < 0.001), and Gdnf (*p* < 0.01), compared to the same treatment in WO cultures (Figure 4A–I, respectively, and Table 1).

Also, the addition of TES to HYP-treated cultures did not affect the expression levels of transferrin and significantly increased the expression levels of Fsh-r (*p* < 0.01) and inhibin b (*p* < 0.001), but significantly decreased the expression levels of Abp (*p* < 0.01), Ar (*p* < 0.05), and the growth factors Csf-1 (*p* < 0.01), Lif (*p* < 0.01), Scf (*p* < 0.05), and Gdnf (*p* < 0.001), compared to the same treatment in WO cultures (Figure 4A–I, respectively, and Table 1). The addition of both FSH and TES (F + T) to HYP-treated cultures did not affect the expression levels of Fsh-r and Abp, but significantly increased the expression levels of inhibin b (*p* < 0.01), and significantly decreased the expression levels of transferrin (*p* < 0.05), Ar (*p* < 0.01), and the growth factors Csf-1 (*p* < 0.01), Lif (*p* < 0.01), Scf (*p* < 0.01), and Gdnf (*p* < 0.001), compared to the same treatment in WO cultures (Figure 4A–I, respectively, and Table 1).

## 3. Discussion

In this work, we evaluated the effect of a significant reduction in/absence of testicular germ cells on the activity of Sertoli cells from immature mice under in vitro culture conditions in the presence and absence of hormones (testosterone and FSH). This condition may simulate unexplained Sertoli cell-only syndrome and pathological conditions such as chemotherapy and radiotherapy treatment administered to prepubertal boys that may cause sterility in their adulthood. Therefore, in the present study, we examined the expression levels of key factors associated with Sertoli cell activity, including Abp, Ar, inhibin b, transferrin, and growth/differentiation factors that are known to be important for germ cell development such as Scf, Gdnf, Lif, and Csf-1 [7,9,10,11,12,13]. Our findings showed that under “normal” (in vitro) conditions, the cultures consisted mainly of Sertoli cells that adhered to the plates, as they could be identified by their characteristic shape and positive staining for vimentin. On the other hand, other somatic cells such as peritubular cells could be present in these cultures and express some of the examined factors. We also reported the presence of germ cells that stained positive for their specific marker VASA. However, after hypotonic shock treatment (causing depletion of germ cells), the adherent cells, mainly Sertoli cells, remained, while most of the VASA-positive cells disappeared. We examined the functionality of Sertoli cells in the culture (in the presence and absence of germ cells and hormones). Our findings showed the expression levels of all examined Sertoli cell functionality markers and growth factors even without hormones. These findings may indicate partial preservation of the basic activity of Sertoli cells in vitro (autoregulation). On the other hand, it should be mentioned that some factors such as Scf, Gdnf, Lif and Csf-1 are also produced by other testicular somatic cells that may present in the culture [9,10,11,12].

Testosterone and FSH are well-known hormones that are involved in the regulation of Sertoli cell activity and spermatogenesis and are involved in the expression of the examined factors under in vivo and in vitro conditions [3,5,7,8,9,10,11,17,32]. Our in vitro results show that the addition of FSH to Sertoli cell cultures (without hypotonic shock; WO) increased the expression levels of transferrin and Fsh-r and decreased the expression levels of Ar and inhibin b without an effect on Abp compared to the control. Furthermore, FSH increased the expression levels of Lif, Scf, and Csf-1 but decreased the expression of Gdnf. These results are in harmony with published studies [7,8,9,10,11], but not in the case of Abp and Gdnf. This could be related to our culture conditions including the time of culture compared to other reports. Studies conducted on Sertoli cell cultures found that an increase in FSH receptors (FSHRs) caused an increase in the proliferation of Sertoli cells [28]. FSH regulates several Sertoli cell activities, such as ABP, GDNF, SCF, growth factor of the insulin family, activin, cytokines, androgens, and their proliferation, differentiation, and apoptosis [8,9,10,11,32,33,34]. These hormones and growth factors are involved in pathways such as cAMP/PKA, ERK1/2, PI3K/Akt, and mTORC1/p70SK6 [8,9,10,28,35].

The addition of testosterone (T) to our cultures significantly increased the expression levels of transferrin, Fsh-r and Abp, but decreased the expression of Ar and inhibin compared to the control. However, testosterone did not affect the expression of the examined growth factors compared to the control. Testosterone can interact with Sertoli cells through several pathways. The conventional mechanism of intravascular T action involves the induction of calcium currents via AR on Sertoli cells leading to CaM/CaMKIV/activation. Another mechanism includes the activation of intracellular signaling through the SHBG (SHBGR) receptor complex (sex hormone-binding globulin) on the membrane of Sertoli cells. Testosterone diffusing into Sertoli cells binds to AR present in the cytoplasm and nucleus to activate the functional responses required to support spermatogenesis [17,36].

The addition of both testosterone and FSH caused an increase in the expression level of Abp, and a decrease in the expression level of inhibin b without any significant change in Fsh-r, Ar, and transferrin expression levels. Overall, a different response of Sertoli cells to the different tested hormones was observed, in response to each of the hormones or both together. These findings could be due to different regulations on the Sertoli cells’ mechanism to produce different factors in culture. Furthermore, the presence of the two hormones together may activate different mechanisms/pathways that may change the effect of each hormone separately. One difference is that testosterone does not regulate cAMP production in Sertoli cells. Another possible difference between FSH and testosterone is related to the regulation of the PI3-K and PLA2 pathways. Additional differences between FSH and testosterone are the up-and-down-regulation of gene expression through AR–DNA interactions that may be achieved only after testosterone stimulation [6].

Hypotonic shock treatment (HYP) of the cultures significantly increased the expression levels of all examined factors compared to WO treatment. These results suggest the crucial role of germ cells in the regulation of Sertoli cell functionality by different mechanisms [10,11,18,19,31].

The addition of FSH to hypotonic shock (HYP) cultures caused a significant decrease in the expression levels of Fsh-r, Abp and caused a significant increase in the expression level of inhibin b without a significant effect on the expression level of transferrin and Ar compared to the same treatment in control cultures (WO). In contrast, the addition of testosterone to HYP cultures caused a significant reduction in the expression levels of Ar, and Abp, but significantly increased the expression levels of Fsh-r and inhibin b. However, it did not significantly affect the expression levels of transferrin compared to the same treatment in control cultures (WO). The addition of both hormones together (testosterone and FSH) caused a significant decrease in the expression levels of transferrin and Ar, and increased the expression level of inhibin b without effect on the expression levels of Fsh-r and Abp after 24 h of culture compared to the same treatment in control cultures (WO). Thus, the addition of FSH and or testosterone decreased some of the Sertoli cell-specific markers and returned them to normal levels.

Furthermore, the addition of FSH and or testosterone to HYO cultures caused a significant decrease in the expression levels of all the examined growth factors such as Gdnf, Scf, Lif and Csf-1 compared to the same treatment in control cultures (WO). Thus, hypotonic shock treatment that may simulate pathology conditions, such as germ cell depletion as a result of chemo-/radiotherapy and induce Sertoli cells to secrete excessive levels of growth factors, and the addition of testosterone and or FSH may restore some Sertoli cell’s functionality to normal.

These findings may suggest that Sertoli cells that undergo hypotonic treatment conditions continue to be active and that the change in their activity is pronounced not only in comparison to the control group but also in response to hormones. This change (depletion of germ cells) violates the balance and relationship between the secreted factors under normal conditions. This type of response probably involves/causes the dysfunction of Sertoli cells interrupts the physiological interactions with germ cells, and probably eventually causes the defect/lack of development of normal spermatogenesis [9,10,11,19].

The contact between germ cells and Sertoli cells is important. Studies show that the primary endocrine control regulation of spermatogenesis through FSH and testosterone is expressed through Sertoli cell activity. Sertoli cells support germ cell development, but one of the most important functions of Sertoli cells is the regulation of the intratubular and intercellular tight junctional environment [37]. The relationship between the germ cells and Sertoli cells is mandatory in the development of the testes as well as in the process of spermatogenesis. In addition, Sertoli cells provide critical factors necessary for the successful progression of germ cells to spermatozoa [18,19,20].

In conclusion, the findings of this work indicate the importance of the interaction between germ cells and Sertoli cells for the continuity of the normal spermatogenesis process. Any damage to germ cells that reduces their number may lead to Sertoli cell dysfunction, ultimately disrupting normal sperm production and potentially impairing male fertility. In addition, our results clearly demonstrate the possible role of FSH and T in maintaining, at least partially, the normal activity of Sertoli cells following germ cell depletion in vitro by hypotonic shock treatment. These results may be relevant to in vitro male fertility preservation/restoration, particularly in certain Sertoli cell-only syndrome patients who still retain a limited number of spermatogonial cells in their seminiferous tubules.

## 4. Material and Methods

### 4.1. Animals

This study was conducted by the Ben-Gurion University Ethics Committee for Animal Use in Research (NO.21-03-2020). Hsd:ICR (CD-1^®^) (ICR; Institute of Cancer Research) and immature mice were used (7-day-old mice) (Harlan Laboratories, Jerusalem, Israel).

### 4.2. Chemicals and Reagents

Dulbecco’s Modified Eagle’s Medium (DMEM), penicillin, streptomycin, and fetal bovine serum (FBS) were purchased from Beit HaEmek Biological Industries (Beit HaEmek, Israel). Collagenase IV (from clostridium histolyticum) and DNAase were obtained from Sigma (St. Louis, MO, USA).

### 4.3. Sertoli Cell Isolation and Hypotonic Shock Treatment

Cells were enzymatically isolated from seminiferous tubules of 7-day-old mice according to our previous study [12]. The cells were suspended in DMEM and FBS (10%) and cultured in 24 well plates (2 × 10^5^ cells/0.5 mL/well). The cells were grown for 3 days for adhesion of the Sertoli cells until a uniform monolayer was formed in the wells. Some of the wells were treated with hypotonic shock, according to our previous study [38]. Thereafter, we added fresh media. After overnight, the media were removed, and fresh media with or without FSH (7 IU/mL) (Merck, Serono, Italy), testosterone (10^−7^ M) (Bayer, Hod Hasharon, Israel), or both together were added. Some of the cells were cultured on coverslips inside the wells under the same treatments. After 24 h of incubation, RNA was extracted from the cultures to be examined for the expression level of Sertoli cell functionality markers, and growth factors by qPCR analysis. The other cultures with coverslips were used to identify the type of cells (Sertoli cells and pre-meiotic cells) (to show that germ cells were depleted following hypotonic shock treatment) that were present in the wells, by specific immunofluorescence staining.

### 4.4. Immunofluorescence Staining

Cells were stained with a primary antibody, i.e., the rabbit monoclonal antibody anti-VASA (D10C5) (Cell Signaling Technology, Danvers, MA, USA). Sertoli cells were stained with a primary antibody, i.e., the monoclonal mouse anti-mouse Vimentin (Sc-373717) (Santa Cruz; 1:100) (Santa Cruz Biotechnology, Inc., Santa Cruz, CA, USA). Slides were incubated overnight at 4 °C. The next day, we added a secondary antibody for 1 h as follows: (donkey anti-mouse IgG (Cy3) and Alexa-flour 488; Jackson Immuno Research (West Grove, PA, USA). Then, we added DAPI that stained the nuclei blue (Santa Cruz Biotechnology, Inc., Santa Cruz, CA, USA).

Microscope: We used the Olympus IX70 microscope (Olympus, Novato, CA, USA).

### 4.5. Calculation the Percentages of VASA-Positive Cells

For untreated cultures (WO) stained for VASA and Vimentin, we counted six different fields, totaling 192 cells. For cultures subjected to hypotonic shock (HYP) and stained for VASA and Vimentin, we counted eight different fields, totaling 194 cells. The percentage of VASA-positive cells was calculated for each counted field.

### 4.6. RNA Extraction and qPCR Analysis:

We extracted the RNA from the in vitro culture as described previously [12] by using the GenElute Mammalian Total RNA Miniprep Kit (Sigma, St. Louis, MO, USA). The RNA was transformed into CDNA by using the qScript cDNA Synthesis Kit (Quantabio, Beverly, MA, USA) and we checked Sertoli cell activity primer markers and Sertoli cell growth factor primer markers (Table 2). The reaction was performed by using qPCR assay. This reaction contained SYBR Green mix (ABgene House, Epsom, UK), DNA polymerase, dNTPs, and dUTP.

Primer sequencing is outlined in Table 1. Our results are expressed as fold increases according to GAPDH. In Figure 2, the ΔΔCt values represent the expression levels of the examined factor relative to GAPDH within each sample.

### 4.7. Data Handling and Statistical Evaluation

All experiments were repeated 3–4 times. All data are expressed as the mean ± SEM (standard error of the mean). Statistical significance analysis was performed with unpaired Student’s *t* tests using prism 9.3.1 (GraphPad software, San Diego, CA, USA). The *p* values below 0.05 were considered significant.

## Figures and Tables

**Table 1:** A summary of the significant effects of hypotonic shock and hormones on the expression levels of factors produced/expressed by Sertoli cells in vitro compared to untreated cultures.

Specific Factors of Sertoli Cells							Growth Factors			
		Transferrin	Inhibin	Ar	Abp	Fsh-r	Csf-1	Lif	Scf	Gdnf
Treatment										
CT		↑	↑	↑	↑	↑	↑	↑	↑	↑
FSH		≠	↑	≠	↓	↓	↓	↓	↓	↓
Testosterone		≠	↑	↓	↓	↑	↓	↓	↓	↓
T+FSH		↓	↑	↓	≠	≠	↓	↓	↓	↓

↑: Indicates an increase, ↓: Indicates a decrease, ≠: Indicates no change.

**Table 2:** Primers for the genes examined for RNA expression by qPCR analysis.

GDNF	Fw-5′-GCCCCTGCTTTCTATCTGCT Rw-5′-AGCCTTCTGAATGCGTGGTT
CSF-1	Fw-5′-CCCATATTGCGACACCGAA Rw-5′-AAGCAGTAACTGAGCAACGGG
LIF	Fw-5′-TAGCGGCTTCAGAAGGGAAAT Rw-5′-AAGGAAAGGAAAGAGGGAGAGC
SCF	Fw-5′-TGAGCCCTTATGCCACACAAT Rw-5′-AAGATGATCCCAAACGCTCGT
Transferrin	Fw-5′-CCAAGCTCCAAACCATGTTGT Rw-5′-ACAGATTGCATGTACTCCGCT
FSH-R	Fw-5′-GTGCATTCAACGGAACCCAG Rw-5′-AGGGAGCTTTTTCAAGCGG
ABP	Fw-5′-GCAGCATGAGGATTGCACTA Rw-5′-CATGAGGCTGGGGAATGTCT
AR	Fw-5′-TTGGGTGTGGAAGCATTGGA Rw-5′-TGGCGTAACCTCCCTTGAAA
Inhibin b	Fw-5′-CCTGTCATCAGGGCAAGTGA Rw-5′-TCGAGGCAGACGCCTTATTC

## Data Availability

The data that support the findings of this study are available from the corresponding author upon reasonable request.
